# Quantitative CT lumbar spine BMD cutpoint value for classifying osteoporosis among older Chinese men can be the same as that of older Chinese women, both much lower than the value for Caucasians

**DOI:** 10.1007/s00256-024-04722-3

**Published:** 2024-06-21

**Authors:** Yì Xiáng J. Wáng, Wing P. Chan, Wei Yu, Ali Guermazi, James F. Griffith

**Affiliations:** 1 Department of Imaging and Interventional Radiology, Faculty of Medicine, The Chinese University of Hong Kong, Shatin, New Territories, Hong Kong SAR, China; 2 Department of Radiology, Wan Fang Hospital, Taipei Medical University, Taipei, Taiwan; 3 Department of Radiology, School of Medicine, College of Medicine, Taipei Medical University, Taipei, Taiwan; 4 Department of Radiology, Peking Union Medical College Hospital, Chinese Academy of Medical Sciences & Peking Union Medical College, Beijing, China; 5 Department of Radiology, VA Boston Healthcare System, Boston University School of Medicine, Boston, MA USA

**Keywords:** Osteoporosis, Bone mineral density (BMD), lumbar spine, Quantitative CT, Chinese men

## Abstract

For older Caucasian women and men, the QCT (quantitative CT) lumbar spine (LS) bone mineral density (BMD) threshold for classifying osteoporosis is 80 mg/ml. It was recently proposed that, for older East Asian women, the QCT LS BMD value equivalent to the Caucasian women’s threshold of 80 mg/mL is about 45∼50 mg/ml. For a data of 328 cases of Chinese men (age: 73.6 ± 4.4 years) who had QCT LS BMD and DXA LS BMD at the same time and with the DXA BMD value of ≤ 0.613 g/cm^2^ to classify osteoporosis, the corresponding QCT LS BMD threshold is 53 mg/ml. Osteoporotic-like vertebral fracture sum score (OLVFss) ≤ -2.5 has been proposed to diagnose osteoporosis. For 316 cases of Chinese men (age:73.7±4.5 years), OLVFss ≤ -2.5 defines an osteoporosis prevalence of 4.4%; to achieve this osteoporosis prevalence, the corresponding QCT LS BMD value is < 47.5 mg/ml. In the China Action on Spine and Hip Status study, a Genant grades 2/3 radiographic ‘osteoporotic vertebral fracture’ prevalence was 2.84% for Chinese men (total n = 1267, age: 62.77 ± 9.20 years); to achieve this osteoporosis prevalence, the corresponding BMD value was < 42.5 mg/ml. In a study of 357 Beijing older men, according to the clinical fragility fracture prevalence and femoral neck DXA T-score, the QCT LS BMD value to classify osteoporosis was between 39.45 mg/ml and 51.38 mg/ml. For older Chinese men (≥ 50 years), we recommend the cutpoint for the QCT LS BMD definition of osteoporosis to be 45∼50 mg/ml which is the same as the value for Chinese women.

Osteoporosis is a systemic skeletal disease characterised by a reduction in bone mass (measured by bone mineral density: BMD) and qualitative skeletal changes that cause an increase in bone fragility and a higher fracture risk. The clinical significance of osteoporosis lies in the fragility fractures (FF) that occur. Numerous studies have demonstrated that the skeleton of East Asians has microstructural and mechanical advantages [1, 2]. For example, Walker et al. [3, 4] reported that postmenopausal Chinese women have a higher trabecular plate-to-rod ratio and greater whole bone stiffness, translating into greater trabecular mechanical competence despite smaller bone size compared to Caucasian women. For the spine, compared with older Caucasians, older Chinese are less likely to have disc space narrowing, thoracic spine hyper-kyphosis, vertebral osteoarthritic wedging, Schmorl nodes defect, and degenerative spondylolisthesis [5, 6]. Compared with British men and women, Japanese men and women were also noted to be less likely to have lumbar spine radiographs osteoarthritic changes graded as Kellgren-Lawrence 4 severity [7]. Compared with Caucasians, East Asians are known to have a lower incidence rate of back pain [5]. Waterman et al*.* [8] queried the National Electronic Injury Surveillance System (USA) for all cases of low back pain presented to emergency departments between 2004 and 2008. They found that the per 1,000 person-years low back pain incident rates were 1.23 among whites, while only 0.20 among Asians. Almost all of the published results comparing East Asians and Caucasians show nearly all FF prevalences, including hip fracture, vertebral fracture, and humerus fracture, are no more than half that of older Caucasians, both for men and women (reviewed in [9, 10]). Figure 1).

**Fig. 1:**
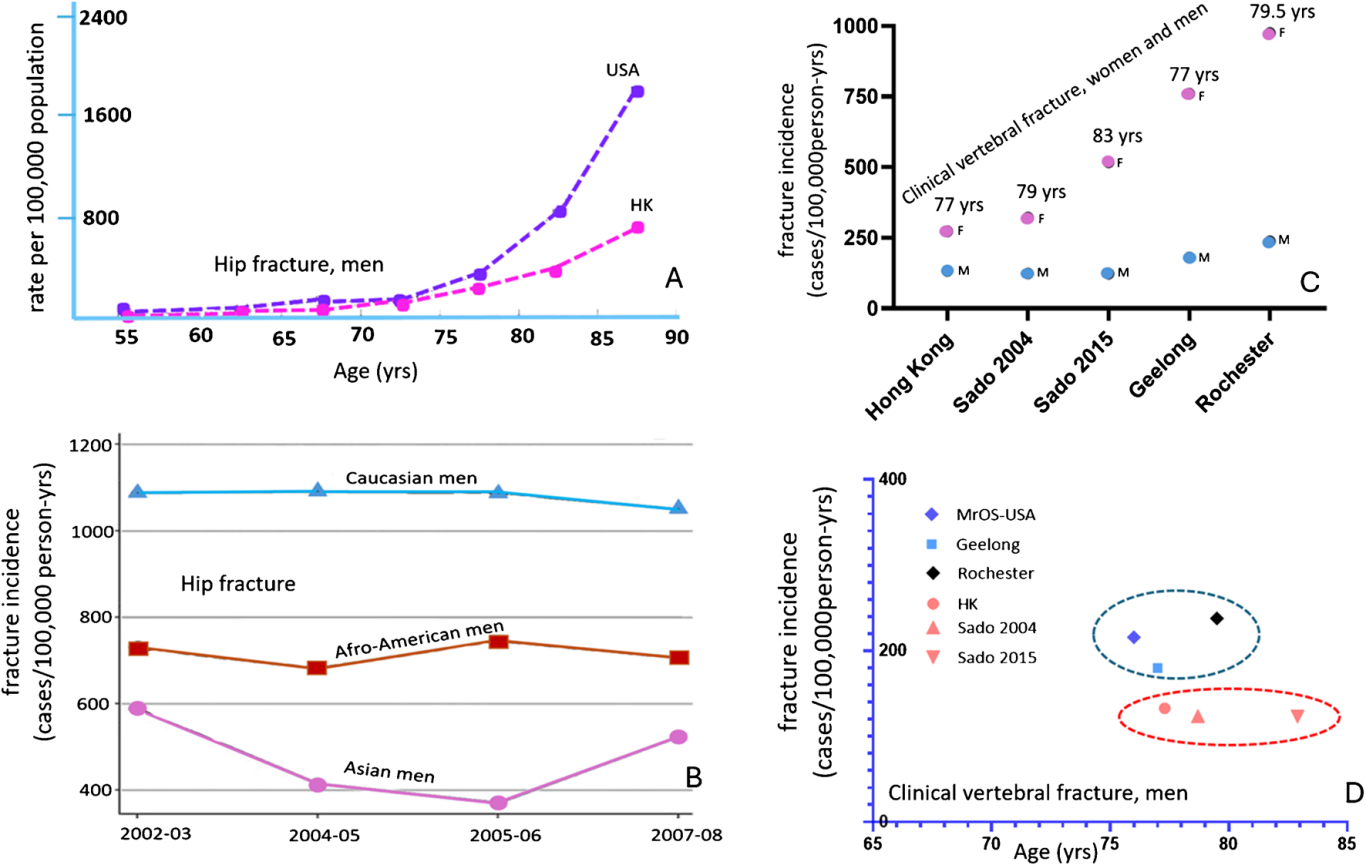
Lower hip fragility fracture (FF) in Asian men relative to Caucasian men (**A, B**), lower clinical vertebral FF in men relative to women (**C**), and lower clinical vertebral FF in East Asian men relative to Caucasian men (**D**). **A**: Hip Fracture Rates in Hong Kong and the United States, 1988 through 1989. Data re-plotted from [17]. **B**: Age-standardized hip fracture incidence in men from 2002 to 2008 for US Medicare beneficiaries (≥ 65 years old). Data re-plotted from Wright et al. J Bone Miner Res 2012;27:2325–32. Data in (**C)** and (**D**) are from ‘Osteoporosis Fracture in Men (Hong Kong)’ study, ‘Osteoporosis Fracture in Women (Hong Kong)’ study, Freitas et al*.* Osteoporos Int 2008;19:615–23 (MrOS USA study), Sanders et al*.* Osteoporos Int 1999;10:240–7 (Geelong study), and Cooper et al*.* J Bone Miner Res 1992;7:221–7 (Rochester study), Sakuma et al*.*, J Bone Miner Metab 2008;26:373–8 (Japan Sado 2004), Imai et al*.*, J Bone Miner Metab 2019;37:484–90 (Japan Sado 2015)

Quantitative CT (QCT) for BMD measurement can be performed on any CT scanner with the use of a calibration phantom. The most common form of QCT provides a trabecular bone measurement [11]. It is expected that the application of QCT for spine BMD will increase, particularly in the settings of opportunistic screening where chest/abdominal CT are conducted for an indication other than osteoporosis. Spine BMD can be simultaneously measured with these chest/abdominal CT scans [12]. For older Caucasian women and men (≥ 50 years), the QCT lumbar spine (LS) BMD cutpoint value for osteoporosis has been recommended to be 80 mg/ml [13, 14]. Instead of applying the Caucasian value of 80 mg/ml, we recently proposed that, for older East Asian women (≥ 50 years), the QCT LS BMD value equivalent to the Caucasian women’s threshold of 80 mg/mL is about 45∼50 mg/ml [15, 16]. FF in men have different features compared with FF in women. FF prevalence in men is no more than half of that in age-matched women (Fig. 1 C, D), this is the same for Caucasian populations and for Chinese population [10, 17, 18]. This article discusses whether QCT LS BMD 45∼50 mg/ml is also a suitable cutpoint value for classifying osteoporosis in older Chinese men.

Firstly, a correlation analysis was conducted with the data of 328 cases of Chinese men (age: 73.6 ± 4.4 years) who had QCT LS BMD and DXA (dual-energy x-ray absorptiometry) LS BMD at the same time. These data are from the Osteoporotic Fracture in Men (MrOS) Hong Kong study year-2 follow-up conducted during the period of September 2003 to March 2005. The result is shown in Fig. 2A. With the Hong Kong local reference, we recommended the DXA LS BMD cutpoint value for classifying osteoporosis in Chinese men to be 0.613 g/cm^2^ (Hologic densitometer) [10, 19]. Following this, Fig. 2A shows the QCT LS BMD threshold for classifying osteoporosis is 53 mg/ml. This result forms the cornerstone of the analyses of the current article, and it is in agreement with other studies such as the mixed-sex cohort study of Chen et al. (Fig. 2B) [20], Lin et al*.* [21], and Uemura et al*.* [22].

**Fig. 2:**
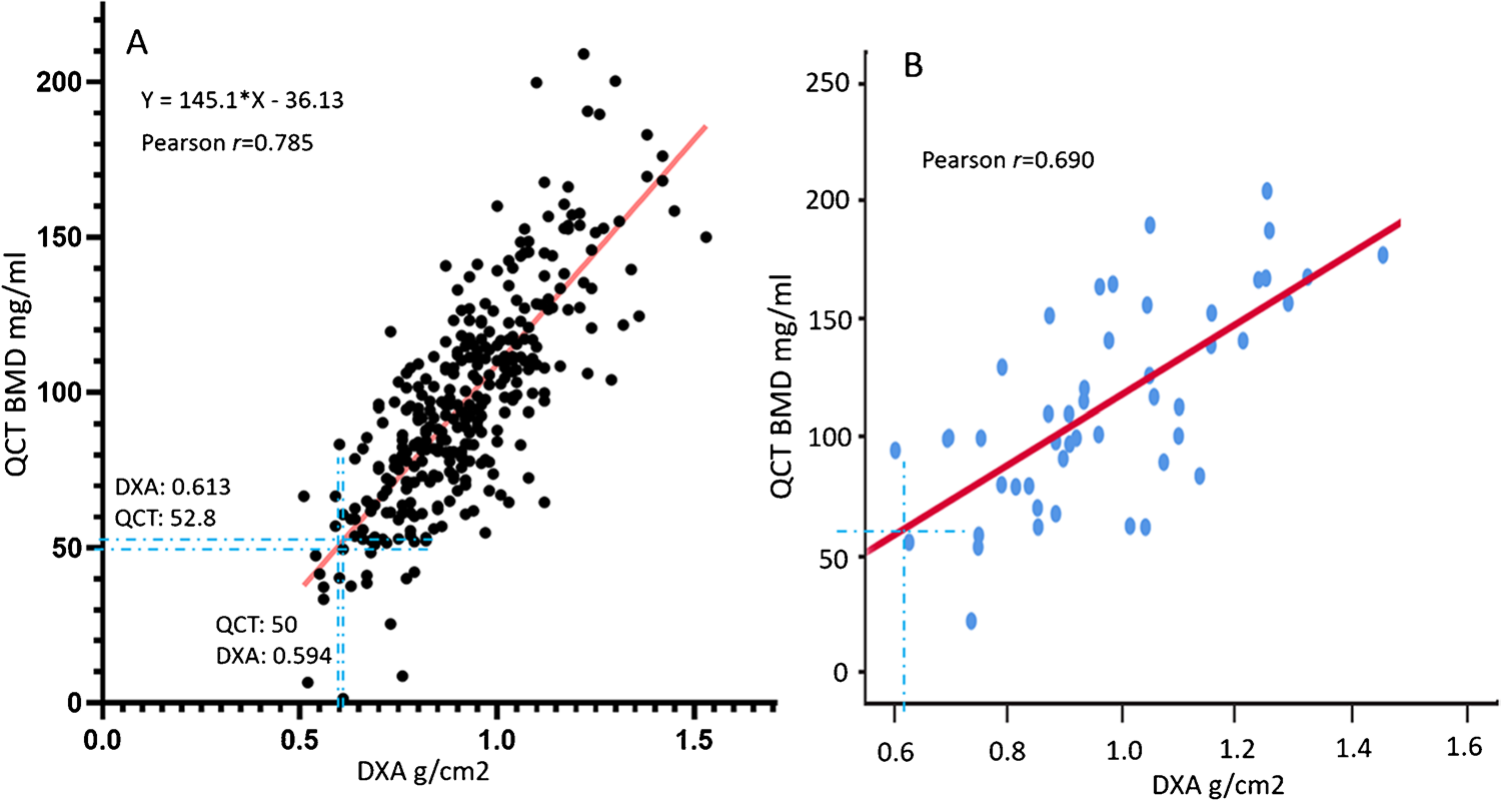
Correlation analyses of LS (lumbar spine) BMD measured by QCT and LS BMD measured by DXA. **A**: 328 Chinese men (age: 73.6 ± 4.4 years) who had QCT LS (L1 and L2) BMD and DXA LS BMD at the same time. The data are from Osteoporotic Fracture in Men (MrOS) Hong Kong year-2 follow-up study. The DXA BMD cutpoint value to define osteoporosis is 0.613 g/cm^2^ (Hologic densitometer) [10], and the corresponding QCT BMD is 53 mg/ml. For QCT value of 50 mg/ml, the corresponding DXA BMD value is 0.594 g/cm^2^. **B**: Results of mixed-sex 48 primary hyperparathyroidism patients (age: 53.77 ± 11.04 years) reported by Chen et al. [20]. The blue dotted crosses show the relationship between DXA measure and QCT measure in (**B**) is approximately similar to that in (**A**)

Secondly, evidence shows QCT LS BMD 45∼50 mg/ml defined osteoporosis is approximately equal to the osteoporosis prevalence defined by radiographic FF or clinical FFs (Table 1). We have recently proposed an index, i.e., osteoporotic-like vertebral fracture sum score (OLVFss), to diagnose osteoporosis [23, 24]. For Chinese men, OLVFss ≤ -2.5 suggests the diagnosis of osteoporosis with the prevalence based on the lowest DXA T-score of femoral neck, total hip, and LS; and among Chinese population the lowest T-score is most likely being the LS T-score [19, 24]. We analysed 316 cases (age: 73.69 ± 4.46 years) of MrOS (Hong Kong) participants with QCT LS BMD and spine radiograph, and the results show OLVFss ≤ -2.5 define osteoporosis prevalence of 4.4% (Fig. 3); to achieve this osteoporosis prevalence, the corresponding QCT LS BMD value is < 47.5 mg/ml. This result is supported by a number of other approximated evidences. Deng et al*.* [25] reported that, for the 70–79 years old group MrOS (Hong Kong) participants (n = 1128), the prevalence of Genant grades 2/3 radiographic ‘osteoporotic’ vertebral fracture is 5.23%. In the CASH (China Action on Spine and Hip Status) study, Liu et al. [26] reported a Genant grades 2/3 radiographic ‘osteoporotic’ vertebral fracture prevalence of 2.84% for their sample (total n = 1267, age: 62.77 ± 9.20 years); and to achieve this osteoporosis prevalence, the corresponding QCT LS BMD value was < 42.5 mg/ml. Yuan et al. [27] studied 357 older men in Beijing, and according to the clinical FF prevalence (i.e., 6.7%) and femoral neck DXA T-score defined osteoporosis prevalence (i.e., 12.3%), the corresponding QCT LS BMD value to classify osteoporosis was between 39.45 mg/ml and 51.38 mg/ml.

**Table 1:** Osteoporosis prevalence among Chinese men or women defined by radiographic fragility fracture (FF) or clinical FF, and the QCT lumbar spine (LS) BMD cutpoint value to define the corresponding osteoporosis prevalence. **a**: For each vertebra, a score of 0, -0.5, -1, -1.5, -2, -2.5, or -3 is assigned for no OLVF (osteoporotic-like vertebral fracture) or OLVF of < 20%, ≥ 20 ~ 25%, ≥ 25% ~ 1/3, ≥ 1/3 ~ 40%, ≥ 40%∼2/3, and ≥ 2/3 vertebral height loss, respectively. OLVFss sums the scores of the T4-L5 vertebrae. OLVFss ≤ -2.5 suggests the diagnosis of osteoporosis according to the lowest T-score of the femoral neck, total hip, and LS (lumbar spine). For Chinese men, the lowest T-score is most likely the LS T-score [24]. QCT was measured at the year-2 follow-up of MrOS Hong Kong study for Chinese men. Half of the corresponding spine radiographs were selected from the baseline study and another half selected from the year-4 follow-up study, since the year-2 follow-up did not have radiograph taken. **b**: while there is no agreed criterion to classify ‘osteoporotic’ vertebral fracture (OVF) among men, Genant semi-quantitative (SQ) grade 2/3 deformities (excluding grade 1) are the most used criteria to diagnose these deformities are ‘osteoporotic’ [Cawthon, et al. Bone 2014;67:152–5]. Still, this may lead to some degree of overestimation for osteoporosis [24], thus BMD cutpoint value should be somewhat lower than the values listed in rows 2, 3. **c**: QCT BMD used MrOS Hong Kong study year-2 follow-up of 328 cases. Osteoporosis result in row 2 broadly supports the result in row 1. **d**: assumed normal distribution of the BMD data. **e**: though Genant SQ grades 1–3 deformities are commonly used criteria to diagnose these deformities are ‘osteoporotic’, it is likely that a portion of grade-1 deformities would be false positive results [Ma and Wang. J Thorac Dis. 2022;14:4685–4698], thus the BMD cutpoint value should be somewhat lower than the value listed in row 4. However, the result of row 4 can support the feasibility of the analyses in rows 2, 3. **f**: excluded subjects with metabolic disorders or usage of drugs which would affect bone metabolism. Clinical FFs likely underestimates the prevalence of densitometric osteoporosis, on the other hand, DXA femoral neck T-score ≤ -2.5 likely overestimates the prevalence of osteoporosis for this sample. For Chinese men, we recommend the cutpoint value for T-score of femoral neck to be -2.7 [19]. Therefore, for this sample, the BMD cutpoint value will be between 39.45 mg/ml and 51.38 mg/ml. Wright et al. [J Bone Miner Res. 2014;29:2520–6] reported that osteoporosis prevalence (either the femoral neck or LS DXA) for Caucasian community men (≥ 50 years) in the US was 3.9%. When correctly measured, the osteoporosis prevalence based on femoral neck and that based on LS will be similar for subjects around 70 years old [Wang. Ann Transl Med. 2022;10:1421], and the osteoporosis prevalence of either the femoral neck or LS will be higher. Though the results of rows 2–6 can be only considered as approximated results, they agree with each other and also agree with the result in row 1, and appear to be reasonable. Data for row 2 is from [25]; data for rows 3, 4 is from [26]; data for rows 5, 6 is from [27]. prev: prevalence. VFF: vertebral FF

data	Mean age:	Criteria to define osteoporosis and the prev	QCT lumbar spine BMD cutpoint value
MrOS Hong Kong yr-2 ^**a, row 1**^	73.7 yrs	OLVFss ≤ -2.5, prev: 4.4% (14/316)	< 47.5 mg/ml defines 4.4% prev
MrOS Hong Kong yr-0 ^**b, row 2**^	75 yrs	Genant SQ grade 2 and 3 VFF, prev: 5.23% (59/1128)	< 51.1 mg/ml defines 5.23% prev ^**c**^
CASH study men ^**b, row 3**^	62.8 yrs	Genant SQ grade 2 and 3 VFF, prev: 2.84% (36/1267)	< 42.5 mg/ml defines 2.84% prev ^**d**^
CASH study women ^**e**, **row 4**^	61.4 yrs	Genant SQ grade 1, 2 and 3 VFF, 15.4% (336/2170)	< 50 mg/ml defines 12.86% prev ^**d**^
Older men in Beijing ^**f**, **row 5**^	74.7 yrs	various clinical FFs, prev: 6.7% (24/357)	< 39.45 mg/ml defines 6.7% prev ^**d**^
Older men in Beijing ^**f, row 6**^	74.7 yrs	DXA femoral neck T-score ≤ -2.5, prev: 12.3% (44/357)	< 51.38 mg/ml defines 12.3% prev ^**d**^

**Fig. 3:**
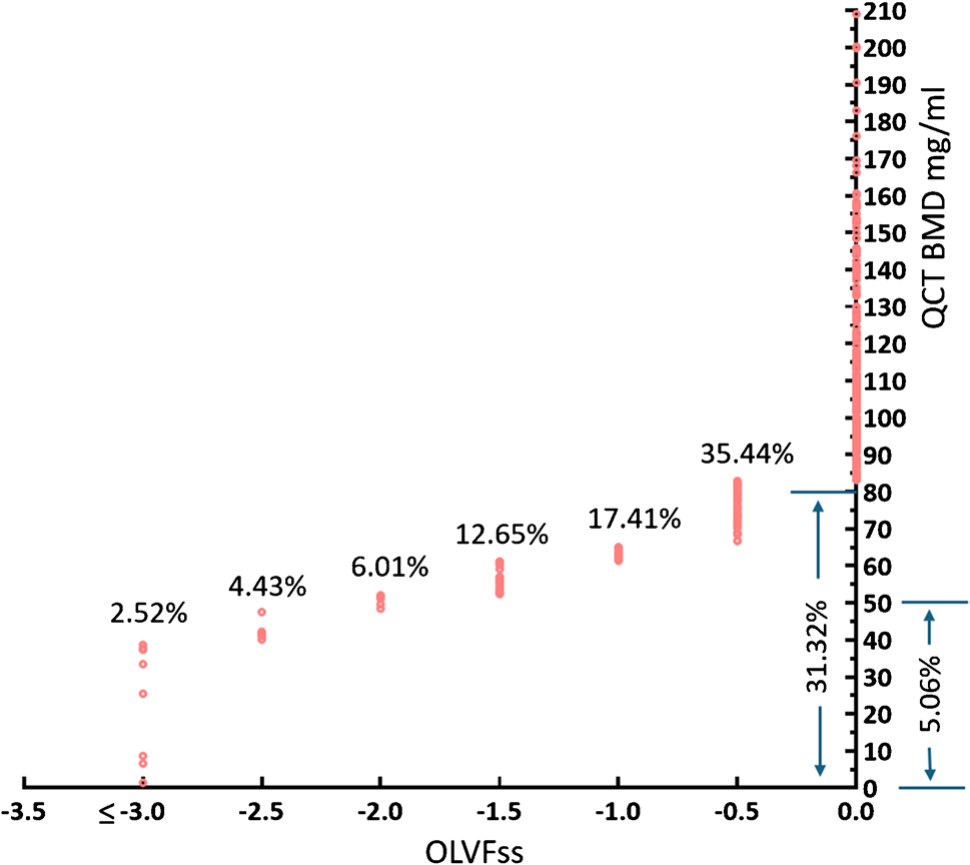
Percentage of osteoporosis defined by OLVFss (osteoporotic-like vertebral fracture sum score) and QCT LS (lumbar spine L1 and L2) BMD. OLVFss ≤ -3, ≤ -2.5, ≤ -2.0, ≤ -1.5, ≤ -1.0, ≤ -0.5 defines 2.52%, 4.43%, 6.01%, 12.65%, 17.41%, and 35.44% of the study subjects (n = 316, mean age: 73.7 years) being osteoporotic, respectively. The recommended OLVFss for defining osteoporosis is ≤ -2.5 [24], and this defines osteoporosis prevalence being 4.43% for this sample. QCT LS BMD of 47.5 mg/ml, 50 mg/ml, 80 mg/ml defines 4.43%, 5.06%, and 31.32% of the subjects being osteoporotic, respectively. Data are from MrOS(Hong Kong) study for Chinese men (Table 1)

There are limitations to the analyses in this article. The main limitation is that analyses of DXA LS BMD and OLVFss in this study are based on the extrapolation (with adjustment according to FF risk profile of Chinese men) of the definition that DXA T-score of Caucasian men for classifying osteoporosis is the same as that of for Caucasian women, i.e. both being -2.5 [10, 24]. There have been some controversies on whether it is appropriate for men and women to share the same osteoporosis diagnosis T-score [28]. However, DXA lumbar spine and femoral neck T-score of Caucasian men for classifying osteoporosis is the same as that of for Caucasian women, i.e. ≤ -2.5, is the currently accepted threshold and has been endorsed by various guidelines [14]. BMD is only one of the contributors to bone strength and bone strength is one of the contributors to fragility fracture risk. Moreover, there is evidence that the association between lower BMD and FF risk is stronger in women than in men [24]. However, a ‘working’ cutpoint value, despite maybe being imperfect, is urgently needed in the current clinical practice.

In conclusion, while there have been fewer results available for men than for women, the data analyses in this article based on DXA LS BMD and QCT LS BMD correlation and supported by the relationship between lower QCT LS BMD and prevalence of fragility fracture consistently suggest that, for older Chinese men (≥ 50 years), the cutpoint for the QCT LS BMD classification of osteoporosis is 45∼50 mg/ml which is the same value as for Chinese women. It is desirable that studies with higher power will be available in the future to further confirm this new osteoporosis cutpoint value. It will be practically convenient if the same cutpoint value is used for both Chinese women and men, i.e., the same as the approach for Caucasians where the same value is applied for women and men. It is likely inappropriate to apply QCT LS BMD 80 mg/ml as a cutpoint value for defining osteoporosis to all ethnic groups worldwide. With the value for Caucasians as the reference, it may be worthwhile to review this cutpoint value for other ethnic groups from Africa and Latin America as well. An ethnic group specific diagnostic criterion will allow a more meaningful comparison of disease burden among different countries, and allow comparable medication intervention thresholds for different ethnic groups.
